# Neurite Outgrowth of Mature Retinal Ganglion Cells and PC12 Cells Requires Activity of CK1δ and CK1ε

**DOI:** 10.1371/journal.pone.0020857

**Published:** 2011-06-16

**Authors:** Joachim Bischof, Adrienne Müller, Miriam Fänder, Uwe Knippschild, Dietmar Fischer

**Affiliations:** 1 Department of General, Visceral and Transplantation Surgery, University of Ulm, Ulm, Germany; 2 Department of Experimental Neurology, University of Ulm, Ulm, Germany; 3 Department of Experimental Neurology, University of Düsseldorf, Düsseldorf, Germany; Florida International University, United States of America

## Abstract

Mature retinal ganglion cells (RGCs) do not normally regenerate severed axons after optic nerve injury and show only little neurite outgrowth in culture. However, RGCs can be transformed into an active regenerative state after lens injury (LI) enabling these neurons to regrow axons *in vitro* and *in vivo*. In the current study we investigated the role of CK1δ and CK1ε activity in neurite outgrowth of LI stimulated RGCs and nerve growth factor (NGF) stimulated PC12 cells, respectively. In both cell types CK1δ and ε were localized in granular particles aligned at microtubules in neurites and growth cones. Although LI treatment did not measurably affect the expression of CK1δ and ε, it significantly elevated the specific kinase activity in the retina. Similarly, CK1δ/ε specific kinase activity was also elevated in NGF treated PC12 cells compared with untreated controls. Neurite extension in PC12 cells was associated with a change in the activity of CK1δ C-terminal targeting kinases, suggesting that activity of these kinases might be necessary for neurite outgrowth. Pharmacological inactivation of CK1δ and ε markedly compromised neurite outgrowth of both, PC12 cells and LI stimulated RGCs in a concentration dependent manner. These data provide evidence for a so far unknown, but essential role of CK1 isoforms in neurite growth.

## Introduction

Neurons of the central nervous system (CNS) are normally unable to regenerate injured axons. This regenerative failure severely limits the chances of recovery after traumatic injuries in the CNS, stroke and in certain neurodegenerative diseases. Reasons for the failure in axonal regeneration are partially due to the insufficient intrinsic capability of adult neurons to regrow axons and to inhibitory factors associated with CNS myelin and glial scar formation [1]–[5]. Mature retinal ganglion cells (RGCs) are typical CNS neurons and possess only weak intrinsic potential to regrow injured axons. However, RGCs are switched into a robust regenerative state when β/γ-crystallins are released from an injured lens [6]–[8]. In this state mature RGCs extend axons in culture at higher growth rates, and regenerate lengthy axons into an injured optic nerve *in vivo* [7]–[10]. Glial derived ciliary neurotrophic factor (CNTF) and leukemia inhibitory factor (LIF) have been identified as the essential mediators of these effects [6], [11]–[16]. However, the molecular processes and regulatory proteins involved in the rearrangement of the cytoskeleton and the regulation of neurite growth in mature RGCs are still poorly understood. Several kinases such as p38 MAPK, ROCK, PKC and PI3K have been identified to regulate axon growth cone stability and guidance [17]–[22]. Studies using RNA interference based screening suggested that approximately 8–9% of the human kinome are involved in promoting or inhibiting neurite outgrowth [23].

Members of the CK1 family comprise a group of ubiquitously expressed second-messenger independent monomeric serine/threonine specific kinases. In mammals seven isoforms (namely CK1α, β, γ_1–3_, δ and ε) and their various splice variants have been described. All CK1 isoforms are highly conserved within their kinase domains, but differ significantly in the length and primary structure of their non-catalytic N-terminal (9–76 aa) and C-terminal (from 24 aa up to more than 200 aa) domains. Within the cell the constitutive phosphotransferase activity of CK1 isoforms is tightly controlled by autophosphorylation, dephosphorylation, proteolytic cleavage and localization to different subcellular compartments [24], [25]. CK1 family members are able to modulate the activity of key regulator proteins involved in several cellular processes such as cell differentiation [26]–[31], proliferation, apoptosis [32]–[36], circadian rhythm [37], chromosomal segregation [38]–[41] and vesicle transport [39], [40], [42]. CK1δ and CK1ε, which share 97% homology within their kinase domains and still exhibit 53% homology within their C-terminal regulatory domains, are able to complement the functions of the CK1 homolog Hrr25 in *Saccharomyces cerevisiae* [43]. Moreover, they exhibit partially overlapping functions in mammals. Both isoforms are highly expressed in the hypophysis, the peripheral nervous system, and the central nervous system [44], [45] and are involved in regulating circadian rhythm [46]. CK1δ has also been reported to regulate dynamics of the cytoskeleton [39], [47]–[50], which is also essential for axonal growth.

Here, we show that CK1δ and ε are expressed in the growth cones of RGCs and PC12 cells and provide evidence that CK1δ and ε activity is essential for neurite growth and extension.

## Results

### Analysis of the expression of CK1δ and ε in the adult retina and PC12 cells

**Figure 1:**
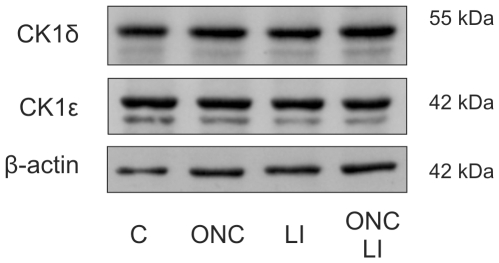
Expression of CK1δ and ε in the retina. Expression of CK1δ and ε in naïve and regenerating retina. Similar levels of CK1δ and ε expression in retinal lysates of untreated animals (C) and rats subjected to optic nerve cut (ONC), lens injury (LI) or ONC+LI were detected by western blot analysis using the CK1δ specific antibody 128A and the CK1ε specific antibody #610446. Detection of β-actin on stripped membranes verified loading of same protein amounts.

In previous reports we have shown a low to moderate expression of CK1δ and CK1ε in the inner nuclear layer (INL) of rat retina and a significantly stronger staining in βIII-tubulin-positive RGCs [44], [45]. In order to test whether CK1δ and ε expression is altered in injured RGCs or when these neurons enter into a regenerative state adult rats were subjected either to an optic nerve cut (ONC) or ONC+lens injury (LI). Neither western blot analysis (Fig. 1), quantitative real-time PCR nor immunohistochemical analysis (data not shown) revealed notable changes in retinal CK1δ and ε expression after ONC or ONC+LI compared with untreated controls, suggesting that expression of CK1δ and ε was neither altered in injured nor regenerating RGCs compared with naïve RGCs.

**Figure 2:**
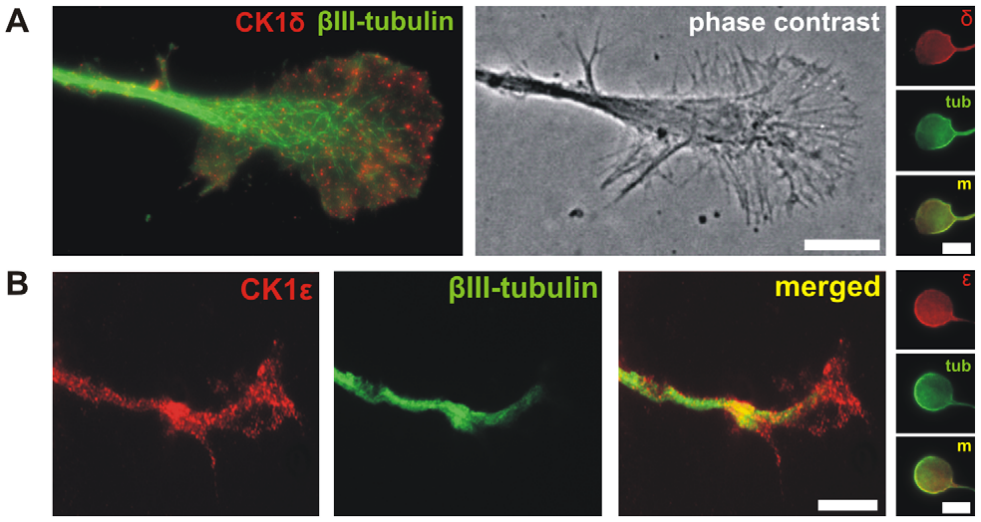
Localization of CK1δ and ε expression in RGCs. (**A**) Immunofluorescence staining and phase contrast image of the neurite growth cone of a RGC using the CK1δ specific monoclonal antibody 128A (red) and a βIII-tubulin specific monoclonal antibody (RB-9249-P0; green). Epifluorescence microscopy of RGCs revealed that CK1δ is located in granular particles aligned at microtubules all over the growth cone. Distribution of CK1δ in the soma is shown in the small vertical image panel (δ: CK1δ, tub: βIII-tubulin, m: merged). Scale bars: 10 µm. (**B**) Co-staining of CK1ε (serum 712; red) and βIII-tubulin (TUJ-1; green) in RGCs revealed a similar expression pattern as shown for CK1δ in (A). Distribution of CK1ε in the soma is shown in the small vertical image panel (ε: CK1ε, tub: βIII-tubulin, m: merged). Scale bars: 10 µm.

To further explore the localization of CK1δ and ε expression in RGCs, we prepared dissociated retinal cell cultures 5 days after ONC+LI. Such *in vivo* pretreated RGCs show spontaneous outgrowth of neurites in culture [15], [51]–[53]. After 48 h in culture cells were fixed for immunofluorescence staining. βIII-tubulin positive RGCs revealed a granular staining pattern of CK1δ and ε along microtubules in the shaft and in the peripheral zones of the growth cones (Fig. 2 A, B). CK1δ and ε were also found to be distributed in the soma of RGCs (Fig. 2 A, B, vertical panel).

**Figure 3:**
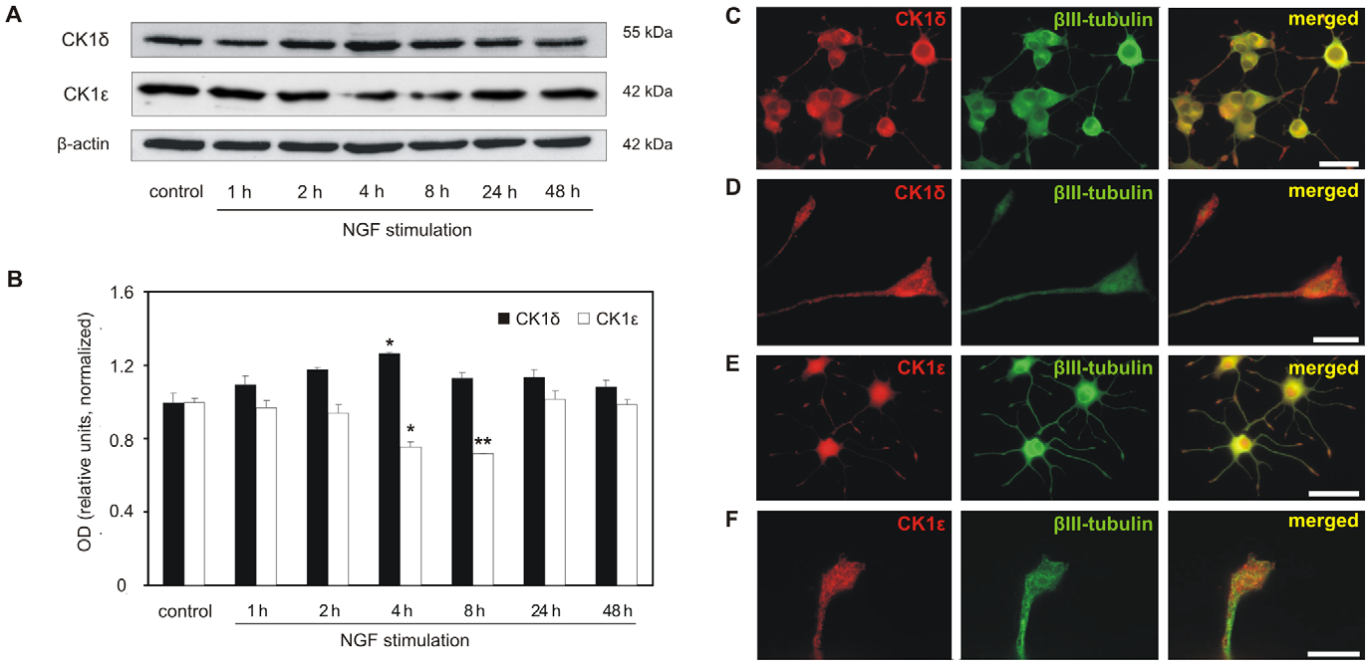
Expression levels and localization of CK1δ and ε in PC12 cells. (**A**) Expression of CK1δ and ε in PC12 cells. CK1δ and ε expression levels were detected in protein lysates of untreated and PC12 cells exposed to NGF for 1–48 h by western blot analyses using CK1δ (128A) and CK1ε (#610446) specific antibodies. β-actin (detected on stripped membranes) served as loading control. (**B**) Densitometric evaluation of western blot results shown in (A). Effects of NGF treatment are significant at * p<0.05 and ** p<0.01 when compared to untreated control cells. (**C, D**) Detection of CK1δ expression in PC12 cells after exposure to NGF for 48 h using antibodies for CK1δ (serum NC10; red) and βIII-tubulin (MAB1637; green). CK1δ positive staining was observed in cell bodies, in neurites and in growth cones (C). Neurite and growth cone are presented at higher magnification in (D). Scale bar for C: 20 µm. Scale bar for D: 2.5 µm. (**E, F**) CK1ε immunostaining in PC12 cells after exposure to NGF for 48 h using antibodies for CK1ε (serum 712; red) and βIII-tubulin (MAB1637; green). CK1ε was detected in the cytoplasm, in neurites and in the growth cones. A magnification of a neurite and growth cone is presented in (F). Scale bar for E: 50 µm. Scale bar for F: 2 µm.

PC12 cells are a commonly used model for studying the molecular mechanisms underlying neurite outgrowth. Therefore we investigated the expression of CK1δ and ε in these cells after neurite outgrowth stimulation by nerve growth factor (NGF). To this end, protein lysates of PC12 cells were prepared from untreated control cells and 1, 2, 4, 8, 24 and 48 h after adding NGF to cell cultures. As determined by western blot analysis and subsequent densitometric evaluation CK1δ expression slightly and transiently increased after 4 h (Fig. 3 A, B). After 8 h the expression returned again to basal levels. Expression of CK1ε significantly decreased 4 and 8 h after NGF stimulation and returned to basal levels after 24 h (Fig. 3 A, B). Immunofluorescence analysis of these cells also showed a distribution of CK1δ and ε in the perinuclear area, in the neurite and the growth cone (Fig. 3 C–F).

### CK1δ/ε kinase activity is increased in differentiating PC12 cells and in the adult retina after LI

**Figure 4:**
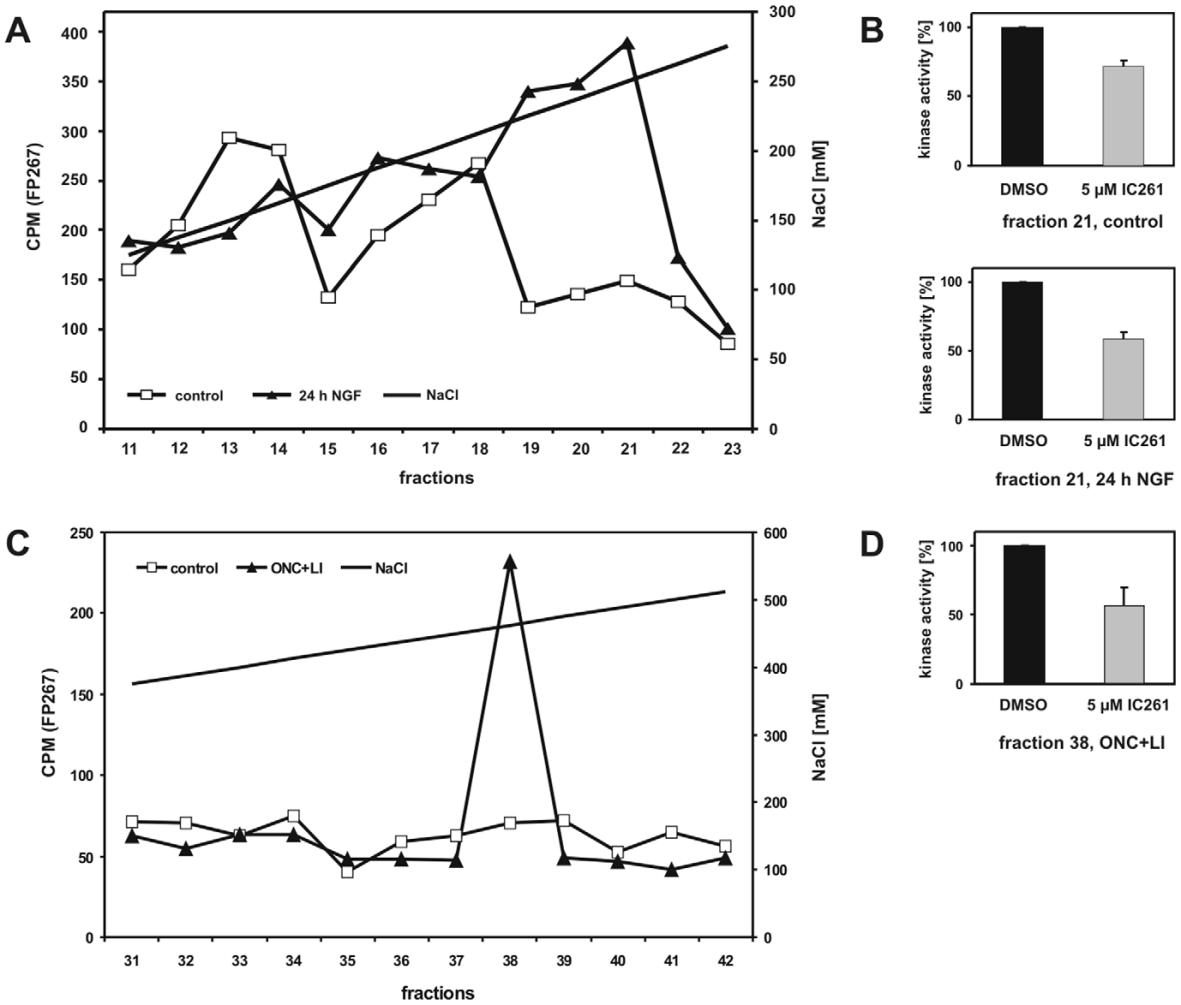
Kinase activities detected in PC12 cells after NGF treatment and in rat retina following ONC+LI. (**A**) Kinase activity in PC12 cells. Equal amounts of protein lysates from PC12 cells either untreated (control) or exposed to NGF for 24 h were loaded onto a 1 ml Resource-Q column for ion exchange chromatography and eluted with a linear gradient of increasing NaCl concentration (represented as a solid diagonal line). Fractions were collected and the CK1δ/ε specific kinase activity was determined in each single fraction as described in detail in Material and Methods. Kinase activities (CPM: counts per minute) are represented by open squares (control) or closed triangles (24 h after NGF stimulation). (**B**) The kinase activity in peak fraction 21 of untreated (control) and NGF stimulated PC12 cells (24 h) was significantly reduced by IC261 specifically inhibiting CK1δ and ε in the micromolar range. Phosphate incorporation into GST-p53^1–64^ (FP267) was quantified by Cherenkov counting. (**C**) Kinase activity in rat retina. Equal amounts of protein lysates from rat retinal tissue either untreated or treated with ONC+LI were loaded onto a 1 ml Resource-Q column for ion exchange chromatography and eluted with a linear gradient of increasing NaCl concentration (represented as a solid diagonal line). Fractions were collected and the CK1δ/ε specific kinase activity was determined in each single fraction as described in detail in Material and Methods. Kinase activities (CPM: counts per minute) are represented by open squares (control) or closed triangles (ONC+LI). (**D**) The kinase activity in peak fraction 38 of extracts prepared from ONC+LI treated animals was significantly reduced by the CK1δ/ε specific inhibitor IC261. Phosphate incorporation into GST-p53^1–64^ (FP267) was quantified by Cherenkov counting.

Although NGF-stimulation of PC12 cells did not affect expression levels of CK1δ and CK1ε we speculated whether it may change the activity of these kinases. This possibility was tested by measuring the specific activity of CK1δ and ε in cell lysate fractions of either untreated or NGF stimulated PC12 cells. One representative result of two independent experiments is shown in Fig. 4 A. GST-p53^1–64^ (FP267) is a well known substrate for CK1 with several potential phosphorylation sites [54]. FP267 was subjected to phosphorylation by aliquots of fractionated protein lysate as described previously [55]. Three major kinase peaks eluting between 130–180 mM NaCl, 200–220 mM NaCl, and 220–250 mM NaCl were detected in lysates of untreated cells (Fig. 4 A). Exposure to NGF for 24 h was followed by a 3-fold increase of the kinase activity in the fraction of the third kinase peak (220–250 mM NaCl) (Fig. 4 A). As shown in Fig. 4 B the presence of IC261, specifically inhibiting CK1δ and ε in the micromolar range, compromised kinase activity of lysate fraction 21 (corresponding to the third major kinase peak), confirming that this fraction mainly represented CK1 activity.

Similar observations were made when kinase activity was measured in fractionated retina protein lysates. No kinase peak was detected in fractionated untreated control lysates using FP267 as substrate (Fig. 4 C). In contrast, ONC+LI treatment induced an increase in kinase activity eluting between 450–475 mM NaCl (fraction 38; Fig. 4 C). Again, the presence of CK1 activity in fraction 38 was confirmed by inhibition of the kinase activity by IC261 (Fig. 4 D).

### NGF treatment changes the activity of cellular CK1δ targeting kinases in PC12 cells

**Figure 5:**
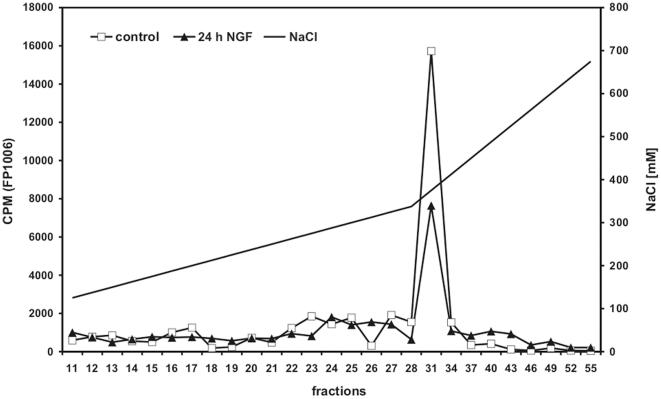
Characterization of CK1δ C-terminal targeting cellular kinase activities. Equal protein amounts of lysates from untreated PC12 cells or PC12 cells exposed to NGF for 24 h were loaded onto a 1 ml Resource-Q column for ion exchange chromatography and eluted with a linear gradient of increasing NaCl concentration (represented as a solid diagonal line). Kinase activity was determined in each single fraction using the GST-CK1δ^305–375^ fusion protein (FP1006) as substrate. Quantification of phosphate incorporation was measured by Cherenkov counting. The major kinase activity eluted at 340–410 mM NaCl in extracts prepared before (open squares) or 24 h (closed triangles) after the induction of differentiation processes of PC12 cells. Data in Fig. 5 show one representative experiment of two.

The activity of CK1δ and CK1ε is modulated by autophosphorylation and by phosphorylation by cellular kinases within the regulatory C-terminal domain [56]–[58]. Thus, changes in the activity of C-terminal targeting kinases upon NGF stimulation of PC12 cells may regulate CK1 activity. To determine the activity of CK1δ C-terminal targeting kinases each fraction of separated protein extracts derived from PC12 cells with or without NGF treatment was used as a source of enzyme. The CK1δ C-terminal fragment GST-CK1δ^305–375^ was used as substrate harboring potential phosphorylation sites for various cellular kinases [57]. As shown in Fig. 5 the major kinase activity (eluting at 340–410 mM NaCl) was reduced by approximately 50% in PC12 cells treated with NGF for 24 h compared to that measured in untreated controls. These results point to the possibility that NGF increased the activity of CK1δ indirectly by reducing the activity of C-terminal targeting kinases.

### CK1-specific inhibitors abolish neurite outgrowth of primary RGCs and PC12 cells

**Figure 6:**
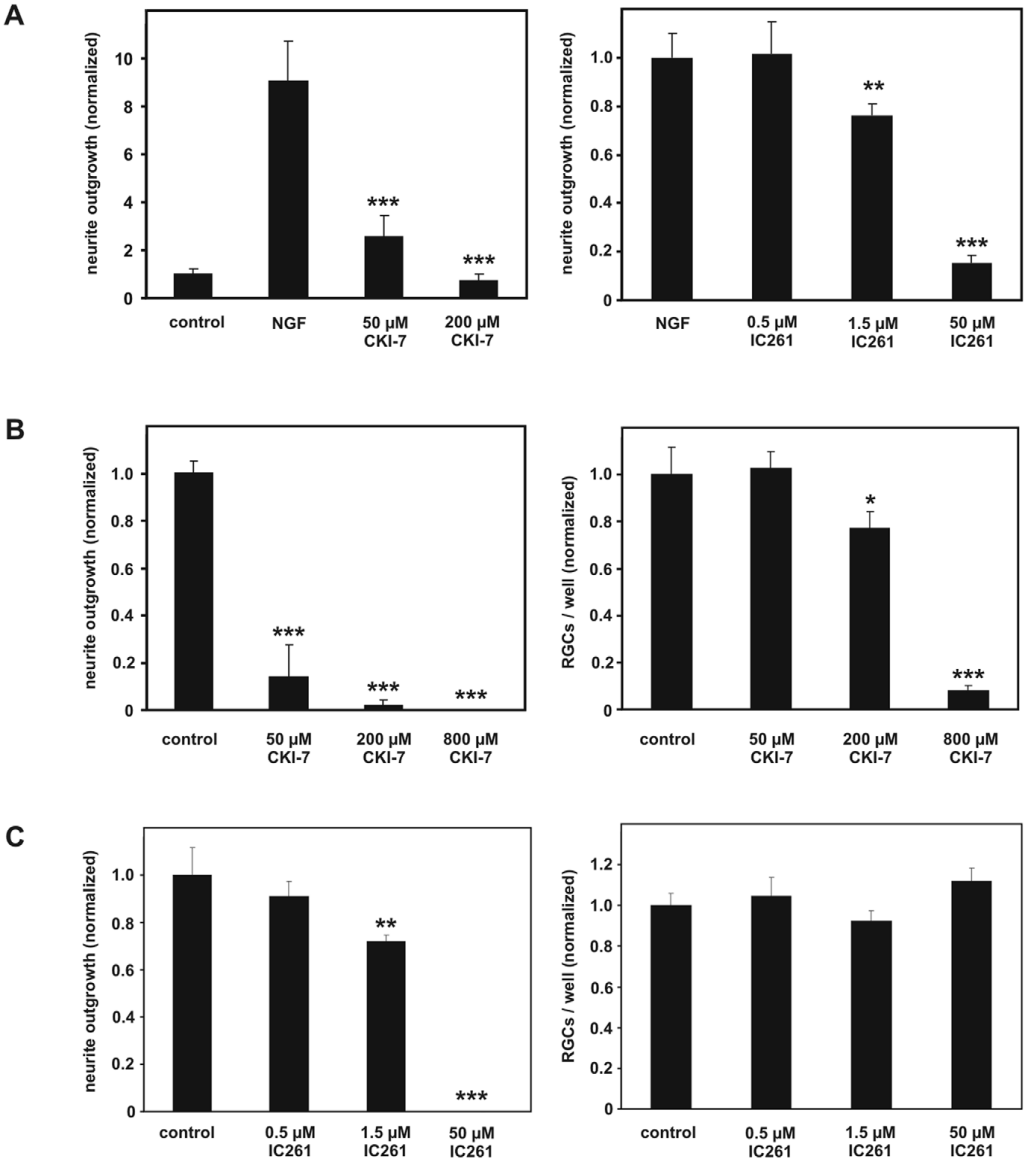
CK1δ and ε specific inhibitors compromise neurite outgrowth of RGCs and PC12 cells. (**A**) PC12 cells were cultured for 3 days without NGF (control) and in the presence of NGF+vehicle or NGF+CKI-7 (50 and 200 µM) or IC261 (0.5, 1.5 and 50 µM). In a concentration dependent manner the average neurite length of NGF stimulated PC12 cells was markedly reduced in the presence of the CK1-specific inhibitors CKI-7 or IC261. Treatment effects: **p<0.01 and ***p<0.001 compared to cells exposed to NGF+vehicle. (**B, C**) LI stimulated RGCs were cultured in the absence (control) or presence of CKI-7 (B; 50, 200 and 800 µM) or IC261 (C; 0.5, 1.5 and 50 µM). Neurite outgrowth was compromised in a concentration dependent manner. The survival of RGCs was only affected in the presence of CKI-7 at 800 µM. Treatment effects compared to control groups: *p<0.05, **p<0.01, ***p<0.001.

Since CK1δ and CK1ε proteins were located in growth cones of RGCs as well as PC12 cells and their kinase activity was increased in growth-stimulated cells, we supposed that CK1δ/ε activity may be involved in neurite outgrowth. To test this possibility we cultured NGF differentiated PC12 cells either in the absence or presence of the CK1-specific inhibitors CKI-7 (50 and 200 µM) or IC261 (0.5, 1.5 and 50 µM). The presence of both inhibitors significantly compromised NGF induced neurite outgrowth in a concentration dependent manner compared with NGF treated PC12 control cells (Fig. 6 A), but did not affect the survival of PC12 cells in culture (data not shown). In primary RGCs that were cultured 5 days after ONC+LI in the presence of 50 µM CKI-7 for 24 h, neurite outgrowth was decreased by 86% compared with controls (Fig. 6 B). When cells were grown in an environment containing 200 µM or 800 µM CKI-7 neurite outgrowth of RGCs was decreased by 98% and 100%, respectively. However, the latter concentrations also significantly affected the survival of RGCs (Fig. 6 B). Moreover, IC261 also significantly compromised neurite outgrowth in a concentration dependent manner (Fig. 6 C). At concentrations of 0.5 µM and 1.5 µM, IC261 significantly reduced neurite outgrowth by 10% and 30%, respectively. Neurite outgrowth was completely abolished in the presence of 50 µM IC261. However, IC261 did not affect the survival of RGCs in culture at the concentrations tested (Fig. 6 C).

### CK1-specific inhibitors destabilize neurite growth cones

**Figure 7:**
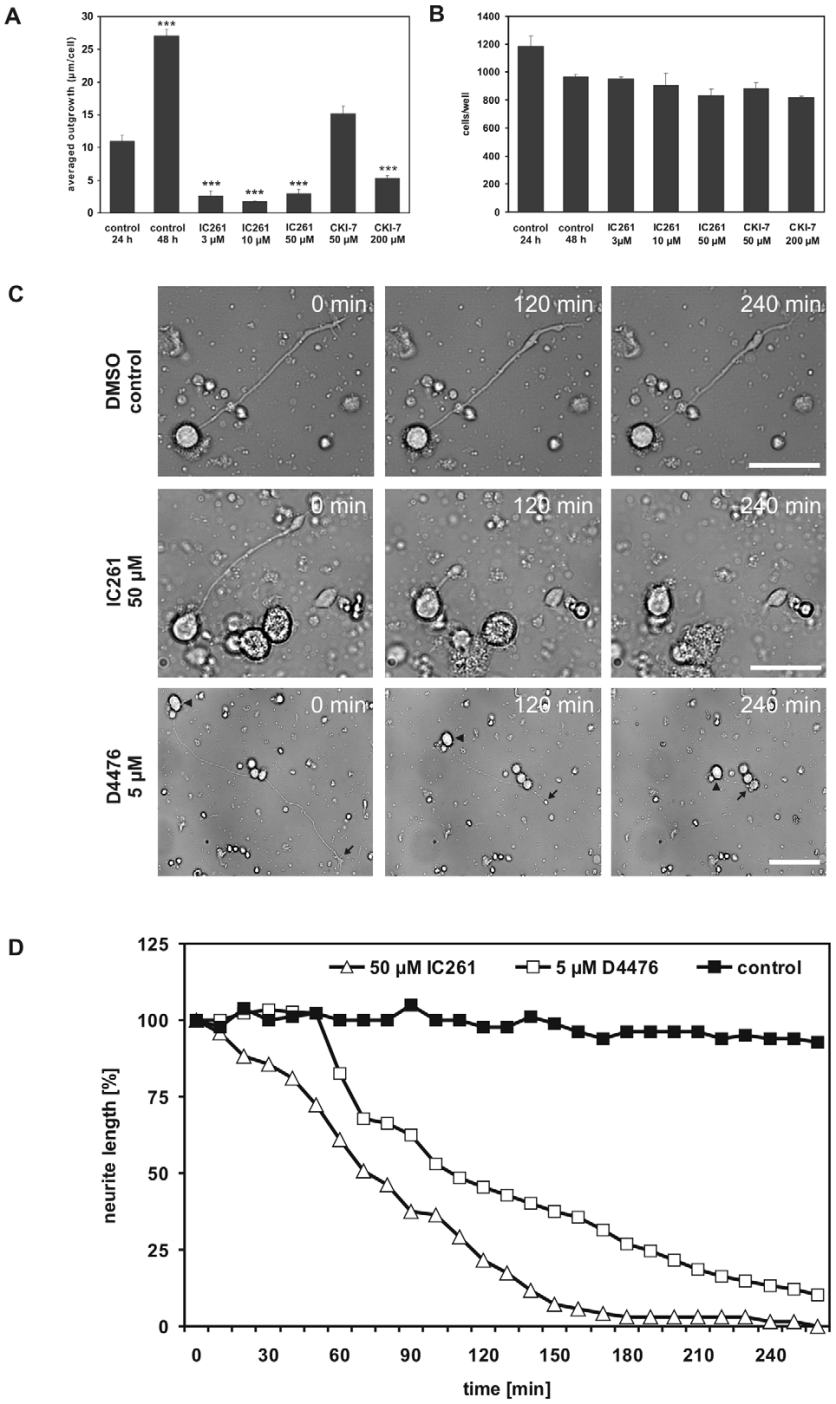
Inhibition of CK1δ and ε destabilizes neurites and growth cones of regenerating RGCs. (**A**) LI stimulated RGCs were cultured for 24 h and then exposed to vehicle or different concentrations of the CK1-specific inhibitors IC261 (0.5, 1.5 and 50 µM) or CKI-7 (50 and 200 µM). The average neurite length of RGCs was determined after 24 h (first control) and 48 h, respectively. Treatment effects: ***p<0.001. (**B**) The number of RGCs per well in all groups was not affected by any treatment described in (A). (**C**) LI stimulated RGCs were cultured for 48 h and subsequently monitored in a flow-through chamber for live cell imaging. In the presence of vehicle or inhibitors IC261 (50 µM) or D4476 (5 µM) cells were recorded for 4 h. Retraction of the outgrown neurite after exposure to IC261 and D4476 is shown at 0, 120 and 240 min. Arrowheads indicate the position of the cell body, arrows the position of the growth cone. Scale bar: 50 µm. (**D**) Quantification of neurite retraction of RGCs over time exposed to vehicle (control, closed squares) or inhibitors IC261 (50 µM, open triangles) or D4476 (5 µM, open squares) specifically inhibiting CK1δ and ε.

Since the CK1-specific inhibitors CKI-7 and IC261 compromised neurite outgrowth further experiments aimed to investigate whether this effect was due to an inhibition of growth cone initiation or to a destabilization of existing growth cones. For this purpose retinal cultures were prepared from animals treated with ONC+LI. After 24 h, when RGCs had already extended neurites, control cells were fixed and others were exposed to vehicle or to increasing concentrations of the CK1-specific inhibitors CKI-7 or IC261. Cultures were incubated for another 24 h prior to fixation. In all tested groups CK1-specific inhibitors did not significantly affect the survival of RGCs (Fig. 7 B). The average neurite length of untreated RGCs averaged 10.9 µm after 24 h and 27.1 µm after 48 h in culture. Cultures treated with CKI-7 or IC261 showed significantly shorter neurites after 48 h than untreated controls after 24 h (Fig. 7 A). These results suggest an essential role for CK1 activity not only for neurite extension, but also for maintaining the stability of the neurite and growth cone, respectively. In order to confirm this hypothesis individual RGCs with a highly dynamic neurite growth cone were recorded by time-lapse microscopy in the absence and presence of either IC261 or D4476, another CK1-specific inhibitor, which blocks CK1δ and ε activity already in the low micromolar range [59]. IC261 (50 µM) induced growth cone collapse and neurite retraction of RGCs (Fig. 7 C). Neurite length was reduced by 50% within 70 min and almost totally retracted after 3 h (Fig. 7 C, D). The cell body of the RGC remained unaffected. Similar results were obtained in the presence of D4476, which induced growth cone collapse and neurite retraction of RGCs at 5 µM (Fig. 7 C, D and video sequence in supporting data file S2). Neurite retraction was almost complete after 4 h, whereas the cell body of the RGC remained unaffected. Control cells were treated with vehicle only (DMSO, transfection reagent; Fig. 7 C, D and video sequence in supporting data file S1).

## Discussion

The main findings of the current study are: (1) CK1δ and CK1ε are localized in neurites and growth cones of PC12 cells and regenerating RGCs, (2) CK1δ/ε activity is increased in differentiating PC12 cells and regenerating retina and (3) CK1-specific small molecule inhibitors destabilize existing growth cones and compromise neurite growth of RGCs and PC12 cells. These observations suggest that the activity of CK1δ and ε is essentially involved and necessary for neurite outgrowth and regeneration.

Cellular kinases and phosphatases are involved in complex signaling during neuronal degeneration and regeneration leading to remodeling of the cytoskeletal architecture [60]–[63]. Members of the CK1 family reportedly play an important role in cytoskeletal rearrangements (reviewed in [25]) by mediating hyperphosphorylation of the microtubule-associated protein tau, which is associated with Alzheimer's disease [48], [64]–[69]. Despite the role of CK1 family members in neurodegenerative disorders, an involvement of these kinases in neurite growth or axonal regeneration has not yet been reported.

Here, we first analyzed the localization of CK1δ and ε in primary, mature RGCs and PC12 cells. Both CK1 isoforms were located in the soma and were aligned in granular structures along microtubules. In addition, we found both isoforms located in the growth cones of regenerating RGCs and PC12 cells. Second we analyzed the occurrence of changes in the expression and activity of CK1δ and ε in regenerating RGCs and differentiating PC12 cells. No notable changes in the expression levels of CK1δ and ε were detected in retinas after optic nerve injury or when RGCs entered into a regenerative state after additional LI. NGF stimulated PC12 cells showed only a slight and temporary upregulation of CK1δ expression and a transient decrease in the expression of CK1ε. However, a 3-fold increase in the CK1δ/ε specific kinase activity was observed in differentiating PC12 cells compared with untreated controls. The CK1δ/ε specific kinase activity was confirmed by its reduction in the presence of small molecule inhibitors specific for CK1δ and ε. These data suggest that the increased CK1δ/ε activity is not basically regulated by the expression levels of CK1δ and ε, but rather through an alternative mechanism. Similar findings were also made in retinal tissue derived from untreated rats and animals that were subjected to ONC+LI. However, CK1δ/ε kinase activities detected in the current study were eluted at a higher NaCl concentration most likely due to changes in the phosphorylation state of CK1δ and ε proteins. Such changes in the chromatographic properties of CK1δ and ε after an isolation from various tissues and cell lines are consistent with previous reports [70], [71]. The activity of CK1δ is reportedly modulated by several cellular kinases, such as PKA, specifically phosphorylating CK1δ within its C-terminal domain [57]. Coherently, we found a reduction in the activity of cellular CK1δ C-terminal targeting kinases in NGF differentiated PC12 cells, suggesting that the increased activity of CK1δ may have been mediated indirectly through altered activity of CK1δ C-terminal targeting kinases. However, additional experiments are necessary to identify this (these) cellular kinase(s) and to characterize their physiological interactions with CK1δ and their role in regulating neurite outgrowth.

Finally, in the current study we demonstrate that pharmacological inhibition of CK1δ/ε activity by two different ATP-competitive small molecule inhibitors markedly compromised neurite outgrowth and induced a destabilization of neurite growth cones of RGCs and PC12 cells. CKI-7 and IC261 effectively blocked neurite outgrowth of regenerating RGCs and PC12 cells at concentrations that did not affect the survival of these cells. Moreover, the fact that all tested CK1δ/ε specific inhibitors, namely IC261, CKI-7 and D4476, exhibited similar results on neurite growth minimizes the possibility of unspecific effects [72]. Thus, the results of the pharmacological inhibition of CK1 imply that CK1δ/ε activity is necessary for neurite outgrowth of primary RGCs. This assumption was further underlined by time-lapse analyses showing that the CK1δ and ε specific inhibitors IC261 and D4476 induced a collapse and retraction of growing neurites of isolated RGCs, probably due to a destabilization of the microtubule and/or actin cable network. This hypothesis is supported by previous observations showing that CK1 is involved in regulating both microtubule and actin filament dynamics [38], [39], [47], [49], [50], [73]–[75].

In order to obtain more information regarding the possible role of CK1 family members in maintaining microtuble integrity their functions in modulating the interaction of α/β-tubulin with microtubule associated proteins (MAPs) should be analyzed more in detail in the future. Additional experiments are also required to clarify the role of CK1δ and ε in regulating actin filament dynamics.

Although the current study suggests that CK1δ/ε activity is required for neurite outgrowth further studies need to be performed to test the possibility as to whether enhancing the activity of CK1δ and ε may facilitate neurite outgrowth and be useful for the development of therapeutic concepts to stimulate axonal regeneration. These experiments may become possible when specific activators either for CK1δ or ε will be available in the future.

## Materials and Methods

### Optic nerve cut and lens injury

All animals were housed and handled in accordance to official regulations for care and use of laboratory animals and maintained under SPF conditions. Ethical approval of all experiments and surgical procedures was approved by the local authorities (Regierungspräsidium Tübingen, permission number 1011).

Adult female Sprague-Dawley rats weighing 220–250 g were anesthetized by intraperitoneal injections of ketamine (60–80 mg/kg) and xylazine (10–15 mg/kg), and a 1–1.5 cm incision was made in the skin above the right orbit. The optic nerve was surgically exposed under an operating microscope, the dural sheath was longitudinally opened and the nerve was cut 1 mm behind the eye, avoiding injury to the central retinal artery. The vascular integrity of the retina was verified by fundoscopic examination. Lens injury (LI) was induced by a retrolenticular approach, puncturing the lens capsule with the tip of a microcapillary tube as described previously [7], [9].

### Isolation of RGCs and tissue culture

Five days after surgical treatment animals were killed by an overdose of chloralhydrate solution (14%) and dissected retinal tissue was incubated in Dulbecco's Modified Eagle medium (DMEM) (Invitrogen, Karlsruhe, Germany) containing papain (16.4 U/ml, Worthington, Katarinen, Germany) and L-cysteine (0.3 µg/ml, Sigma-Aldrich, Munich, Germany) for 30 min at 37°C as described previously [52]. After digestion, the retinal tissue was rinsed twice with DMEM before being transferred into DMEM containing B27-supplement (1∶50, Gibco, Karlsruhe, Germany) and penicillin/streptomycin (0.2 mg/ml, Biochrom, Berlin, Germany). Then, the triturated retina was passed through a cell strainer before seeding of the isolated RGCs onto poly-D-lysine (0.1 mg/ml, molecular weight <300000 Da, Sigma-Aldrich, Munich, Germany) and laminin (20 µg/ml, Sigma-Aldrich, Munich, Germany) coated culture dishes.

PC12 cells [76] were seeded on poly-D-lysine coated tissue culture dishes and maintained in DMEM supplemented with 10% horse serum, 5% fetal calf serum (both Gibco, Karlsruhe, Germany) and penicillin/streptomycin (0.2 mg/ml, Biochrom, Berlin, Germany) at 37°C in a humidified atmosphere (85% humidity) containing 5% CO_2_. In neurite outgrowth assays, cells were treated with NGF (100 ng/ml) as indicated, the CK1-specific kinase inhibitors CKI-7 (Sigma-Aldrich, Munich, Germany) [77] and IC261 (ICOS Corporation, Bothell, USA) [78] were added directly after seeding. In each case neurite outgrowth was quantified using the public domain image processing software *ImageJ* (National Institutes of Health, Bethesda, USA). The significances of intergroup differences were evaluated using a one-way analysis of variance (ANOVA) test, followed by a correction of a post hoc test (Turkey). All data are provided as average and standard error (SEM).

### Time-lapse microscopy of RGCs

For live cell imaging, RGCs were grown on glass slides for 48 h and then transferred to a flow-through chamber for inverted microscopes (Bioptechs, Butler, USA) and further cultivated in supplemented RPMI (Invitrogen, Karlsruhe, Germany; containing B27, 1∶50) at a medium flow-rate of 1 ml/h. After 30 min, medium was exchanged to growth medium containing the CK1-specific inhibitors IC261 (ICOS Corporation, Bothell, USA) [78] or D4476 (Calbiochem, Darmstadt, Germany) [59] or DMSO/transfection reagent (as a negative control). D4476 was applied using transfection reagent (Effectene, Qiagen, Hilden, Germany) as described previously [59]. The cells were monitored for 4 h and phase contrast or bright field time-lapse recordings were taken under 40× magnification using the Olympus IX81 microscope (Olympus, Hamburg, Germany) and the Cell^R^ software.

Neurite length was quantified every 10 min before and during cell treatment using the public domain image processing software *ImageJ* (National Institutes of Health, Bethesda, USA).

### Antibodies

For immunofluorescence staining monoclonal antibodies specific for βIII-tubulin were purchased from Babco (Richmond, USA; TUJ-1, mouse, 1∶2000), Millipore (Billerica, USA; MAB1637, mouse, 1∶1000) and Thermo Fisher Scientific (Fremont, USA; RB-9249-P0; rabbit; 1∶500). For detection of CK1δ the mouse monoclonal antibody 128A (ICOS Corporation, Bothell, USA; 1∶500) and the rabbit polyclonal serum NC10 [39] (1∶200) were used. CK1ε was detected using rabbit polyclonal serum 712 [79] (1∶200). Fluorescence labeled secondary antibodies anti-mouse/anti-rabbit Alexa Fluor 488 or anti-rabbit/anti-mouse Alexa Fluor 633 (each 1∶1000) were supplied by Molecular Probes (Eugene, USA).

In western blot analyses, mouse monoclonal antibodies against CK1δ (128A, ICOS Corporation, Bothell, USA; 1∶5000), CK1ε (#610446, Becton Dickinson, Franklin Lakes, USA; 1∶150), or β-actin (A5441, Sigma-Aldrich, Munich, Germany; 1∶10000) were used. Immunocomplexes were detected using anti-mouse HRP conjugated IgG (both 1∶10000, GE Healthcare, Chalfont St Giles, GB).

### Immunofluorescence microscopy

Cells grown on coated coverslips were washed twice with PBS and fixed in 4% paraformaldehyde for 30 min. Fixed cells were permeabilized using 0.3% Triton X-100 and blocked with 2% BSA in PBS/Tween (0.05%) for 1 h followed by incubation with primary antibodies for 45 min at room temperature or over night at 4°C. After washing with PBS secondary fluorescence labeled secondary antibodies were applied for 30 min at room temperature. Finally, cells were embedded in mounting medium containing 5% polyvinyl alcohol (MW 70000–100000 Da, Sigma-Aldrich, Munich, Germany) and 10% glycerol (Roth, Karlsruhe, Germany) in PBS. Analyses and documentation were done using a fluorescence microscope and a high-resolution digital camera (Olympus, Hamburg, Germany).

### Overexpression and purification of glutathione S-transferase fusion proteins

The production and purification of the GST-fusion proteins FP267 (GST-p53^1–64^) and FP1006 (GST-CK1δ^305–375^) were carried out as described elsewhere [55], [57].

### Fractionation of proteins

Untreated and differentiated PC12 cells were washed with PBS and lysed in sucrose lysis buffer containing 20 mM Tris-HCl [pH 7.0], 0.27 mM sucrose, 1% Triton X-100, 1 mM EGTA, 1 mM benzamidine, 50 µM leupeptin, 1% Trasylol (aprotinin), and 0.1% β-mercaptoethanol (MSH) on ice. Cell lysates were passed through a 0.45 µm filter and in each case 25 mg of total protein were applied to an anion exchange column (Resource-Q 1 ml) attached to an ETTAN LC purifier (both GE Healthcare, Chalfont St Giles, GB). Proteins were eluted with a linear ascending gradient between 0–1000 mM NaCl in 50 mM Tris-HCl [pH 7.5], 1 mM EDTA, 5% glycerol, 0.04% Brij, 1 mM benzamidine, 4 µg/ml leupeptin, 1% Trasylol (aprotinin) and 0.1% β-mercaptoethanol. Fractions of 250 µl volume were collected. Aliquots of 2 µl of each fraction were used for *in vitro* kinase assays to determine kinase activities in single fractions.

### *In vitro* kinase assays

*In vitro* kinase assays were carried out in the absence or presence of the CK1-specific inhibitor IC261 [78] in kinase buffer containing 100 nM ATP, 25 mM Tris-HCl [pH 7.5], 10 mM MgCl_2_, 0.1 mM EDTA and 2 µCi [^32^P] γ-ATP. GST0p53^1–64^ (FP267, [55], [57]) and GST-CK1δ^305–375^ (FP1006, [57]) were used as substrates. Single fractions of fractionated PC12 cell extracts were used as sources of enzyme. Phosphorylated proteins were separated by SDS-PAGE and the protein bands were visualized on dried gels by autoradiography. Phosphorylated protein bands were excised and quantified by Cherenkov counting.

### Western blot analysis

For the detection of CK1δ and CK1ε in retinal tissue of untreated and treated animals and differentiated PC12 cells, respectively, protein lysates were prepared in lysis buffer containing 50 mM Tris-HCl [pH 8.0], 120 mM NaCl, 0.5% NP40, 10% glycerol, 1 mM EGTA, 1 mM benzamidine, 50 µM leupeptin, 1% Trasylol (aprotinin) and 5 mM DTT. Extracts were clarified by centrifugation. 75 µg of each protein extract were separated by SDS-PAGE and transferred onto a PVDF blotting membrane (Hybond P, GE Healthcare, Chalfont St Giles, GB). The membranes were probed with primary antibodies and immunocomplexes were detected using HRP conjugated IgG followed by chemiluminescence detection. Where indicated membranes were stripped before being used for reblotting.

## Supporting Information

Supporting data file S1 **Time-lapse recording of RGCs treated with DMSO/transfection reagent.** After 48 h in culture, dissociated LI stimulated RGCs were monitored in a flow-through chamber for live cell imaging. As indicated, cells were treated with vehicle (DMSO and transfection reagent). Time-lapse recordings were taken for 4 h.
(WMV)

Supporting data file S2 **Time-lapse recording of RGCs treated with 5 µM D4476.** After 48 h in culture, dissociated LI stimulated RGCs were monitored in a flow-through chamber for live cell imaging. As indicated, cells were treated with the inhibitor D4476 (5 µM) specifically inhibiting CK1δ and ε, using transfection reagent (Effectene, Qiagen, Hilden, Germany) as described previously [59]. Time-lapse recordings were taken for 4 h.
(WMV)
